# Economic impact of individualized antimicrobial dose optimization in the critically ill patient in Spain

**DOI:** 10.3389/fphar.2025.1506109

**Published:** 2025-02-26

**Authors:** Santiago Grau, Sonia Luque, Olivia Ferrandez, Adela Benitez Cano, Darío Rubio-Rodríguez, Carlos Rubio-Terrés

**Affiliations:** ^1^ Hospital del Mar, Parc de Salut Mar, Barcelona, Spain; ^2^ Universitat Pompeu Fabra, Barcelona, Spain; ^3^ Health Value, Madrid, Spain

**Keywords:** therapeutic drug monitoring, dose optimization, antimicrobials, critical ill patient, economic impact

## Abstract

**Objective:**

To estimate the economic impact of individualized dose optimization guided by antimicrobial therapeutic drug monitoring (TDM) in Spain, compared to no monitoring.

**Methods:**

A cost analysis of antibiotic treatment of critically ill patients, with and without TDM, was performed using a probabilistic Markov model (with second-order Monte Carlo simulations). Three scenarios were analyzed based on three published meta-analyses (Analysis 1: Pai Mangalore, 2022; Analysis 2: Sanz-Codina, 2023; Analysis 3: Takahashi, 2023).

**Results:**

TDM, compared to the no-TDM option, generated according to the meta-analysis, a per patient expenditure of €195 (95%CI €194; €197) in analysis 1 or savings of -€301 (95%CI -€300; -€304) and -€685 (95%CI -€685; -€684) in analyses 2 and 3. The probability of TDM (vs. no-TDM) generating savings would be 39.4%, 63.5% and 79.7% in analyses 1, 2 and 3, respectively. This discrepancy in the results is due to methodological differences, in particular in the cure rate with TDM (vs. no-TDM) obtained in the meta-analyses: 12.2%, 16.6% and 16.0% more in analyses 1, 2 and 3, respectively.

**Conclusion:**

In critically ill patients undergoing antimicrobial therapy TDM, there is an increased likelihood of cure. However, the currently available data are not conclusive on the economic impact of such a therapeutic effect.

## Highlights

• TDM, compared to the no-TDM option, generated according to the meta-analysis, a per patient expenditure of €195 (95%CI €194; €197) in analysis 1 or savings of -€301 (95%CI -€300; -€304) and -€685 (95%CI -€685; -€684) in analyses 2 and 3.
• The probability of TDM (vs. no-TDM) generating savings would be 39.4%, 63.5% and 79.7% in analyses 1, 2 and 3, respectively.
• This discrepancy in the results is due to methodological differences, in particular in the cure rate with TDM (vs. no-TDM) obtained in the meta-analyses: 12.2%, 16.6% and 16.0% more in analyses 1, 2 and 3, respectively.

## 1 Introduction

Antimicrobial therapeutic drug monitoring (TDM) consists of the determination of their plasma levels, followed by dose adjustment according to the results obtained (Impact on the clinical outcomes and cost-effectiveness of the antimicrobial therapeutic monitoring program in critical patients, 2025). The aim is to achieve therapeutic plasma concentrations that allow an optimal pharmacokinetic/pharmacodynamic (PK/PD) ratio to be achieved and to avoid both sub-therapeutic concentrations that could compromise therapeutic success and supra-therapeutic concentrations that could lead to toxicity (Impact on the clinical outcomes and cost-effectiveness of the antimicrobial therapeutic monitoring program in critical patients, 2025; Nielsen et al., 2011). The ultimate goal is to select the most appropriate dosing regimen for each patient for a given drug according to the pathophysiological conditions of the patient, the type of infection, and the causative agent (Impact on the clinical outcomes and cost-effectiveness of the antimicrobial therapeutic monitoring program in critical patients, 2025) However, the therapeutic benefits of antimicrobial dose optimization based on TDM are unclear, which is why three recent meta-analyses have been published (Pai Mangalore et al., 2022; Sanz-Codina et al., 2023; Takahashi et al., 2023). According to the meta-analysis by Pai Mangalore et al. (2022), which included 11 studies (both observational and randomized clinical trials (RCTs), TDM-guided antibiotic dosing would be associated with a statistically significant increase in clinical cure (relative risk, RR = 1.17, 95%CI 1.04-1.31). Still, no reduction in mortality or length of hospital stay would be observed. According to the meta-analysis by Sanz-Codina et al. (2023), which included 10 RCTs, TDM is associated with a reduction in treatment failure (RR = 0.70, 95%CI 0.54-0.92) but not in mortality (RR = 0.86, 95%CI 0.71-1.05). Finally, according to the meta-analysis by Takahashi et al. (2023), which included 5 RCTs, no statistically significant differences were found in clinical cure rate (RR = 1.23; 95%CI 0.91-1.67) and day 28 mortality (RR = 0.94; 95%CI 0.77-1.14), nor in length of intensive care unit (ICU) stay (mean difference in days = 0; 95%CI -2.18, 2.19). Due to these doubts about the clinical benefits associated with TDM, a cost-minimization analysis was chosen, as the trend of the estimated differences, although not statistically significant, could have an economic impact through possible savings from reduced length of stay in the ICU and costs associated with treatment failures, with the consequent prolongation of hospital stay and need for second-line antibiotic treatment. Consequently, the present study aimed to estimate the economic impact of individualized antimicrobial dose optimization guided by TDM in Spain compared to the lack of monitoring.

## 2 Methods

### 2.1 Economic model

**FIGURE 1:**
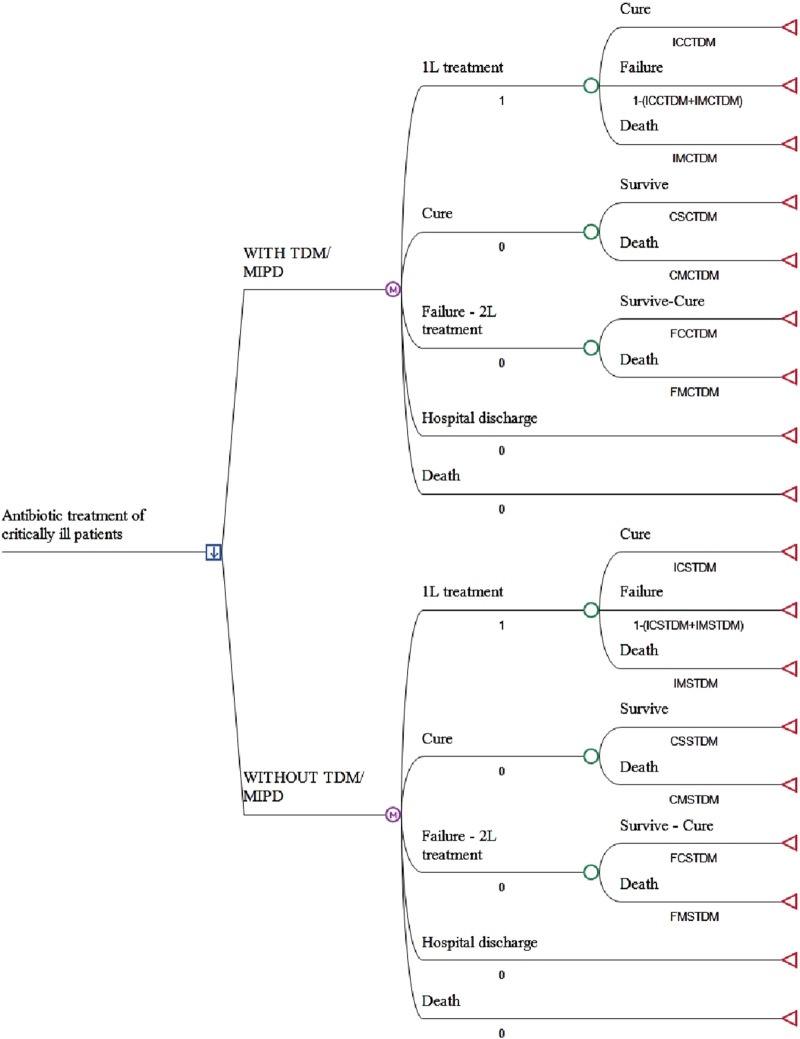
Markov model structure. 1L: firts line of antibiotic treatment; 2L: second line of antibiotic treatment; TDM/MIPD: antimicrobial therapeutic drug monitoring. See the abbreviated meaning of the variables in Table 1.

A cost analysis was performed to evaluate antibiotic treatment of critically ill patients, with and without TDM, using a Markov model. Five health states were considered: first-line treatment (1L) (the state in which the entire patient cohort starts the simulation), cure, second-line treatment-failure (2L), hospital discharge and death (Figure 1). The evolution of patients with and without TDM to the progression states of cure, failure, survival and death was modelled. A static Markov model with a single transition was performed, assuming a median length of hospital stay of 9–14 days in the case of cure and 18–30 days if first-line antibiotic treatment fails, according to the meta-analysis of Pai Mangalore et al. (2022).

A hypothetical cohort of patients admitted to the ICU with suspected or confirmed bacterial infection of respiratory, urological or abdominal focus (critical patients) was modelled, with critical patients understood as those in a clinical situation in which one or more vital functions/systems are altered, thus placing them in potential or actual life-threatening situations (Kayambankadzanja et al., 2022). Deterministic and probabilistic analyses were performed (the latter using second-order Monte Carlo simulations with 1,000 iterations) (Eckhardt et al., 1987). The costs were adjusted to gamma distributions and probabilities to beta distributions. Parameters α and λ of the gamma distributions and α and β of the beta distributions were obtained from the means and standard deviations of each variable using the method of moments (Peral et al., 2020).

Three scenarios were analyzed based on the three published meta-analyses: Analysis 1 (Pai Mangalore et al., 2022); Analysis 2 (Sanz-Codina et al., 2023); Analysis 3. (Takahashi et al., 2023). It is important to highlight that the meta-analysis by Pai Mangalore et al. (analysis 1) only included patients treated with beta-lactam antibiotics, that the meta-analysis by Sanz-Codina et al. (analysis 2) included both beta-lactams and vancomycin and that, finally, the meta-analysis by Takahashi et al. (analysis 3) included aminoglycoside antibiotics in addition to beta-lactams and vancomycin.

For greater clarity on how the economic model works, please see the more detailed (step-by-step) explanation in Supplementary Material S1.

### 2.2 Transition probabilities

Transition probabilities between health states with and without TDM were obtained from the meta-analysis for each analysis (Table 1) (Pai Mangalore et al., 2022; Sanz-Codina et al., 2023; Takahashi et al., 2023). The mortality rate in the general population was obtained from the National Institute of Statistics (www.ine.es).

**TABLE 1:** Transition probabilities of the economic model.

Item	Variable name	Variable description	Average probability	MIN	MAX	SD
Analysis 1 Pai Mangalore et al. (2022)						
WITH TDM	ICCTDM	Critical infection/Cure with TDM	0.6897	0.6207	0.7587	0.0352
	IMCTDM	Critical infection/Death with TDM	0.1778	0.1600	0.1956	0.0091
	IFCTDM	Critical infection/Failure with TDM	0.1325	0.2193	0.0458	0.0443
	CMCTDM	Cure/Death with TDM	0.0035	0.0032	0.0039	0.0002
	CSCTDM	Cure/Survive with TDM	0.9965	0.9969	0.9962	0.0002
	FCCTDM	Failure/Cure with TDM	0.7859	0.7073	0.8645	0.0401
	FMCTDM	Failure/Death with TDM	0.2141	0.2927	0.1355	0.0401
WITHOUT TDM	ICSTDM	Critical infection/Cure without TDM	0.5673	0.5106	0.6240	0.0289
	IMSTDM	Critical infection/Death without TDM	0.2141	0.1927	0.2355	0.0109
	IFSTDM	Critical infection/Failure without TDM	0.2186	0.2967	0.1405	0.0399
	CMSTDM	Cure/Death without TDM	0.0035	0.0032	0.0039	0.0002
	CSSTDM	Cure/Survive without TDM	0.9965	0.9969	0.9962	0.0002
	FCSTDM	Failure/Cure without TDM	0.7859	0.7073	0.8645	0.0401
	FMSTDM	Failure/Death without TDM	0.2141	0.2927	0.1355	0.0401
Analysis 2 Sanz-Codina et al. (2023)						
WITH TDM	ICCTDM	Critical infection/Cure with TDM	0.6370	0.5733	0.7007	0.0325
	IMCTDM	Critical infection/Death with TDM	0.2360	0.2124	0.2596	0.0120
	IFCTDM	Critical infection/Failure with TDM	0.1270	0.2143	0.0397	0.0445
	CMCTDM	Cure/Death with TDM	0.0035	0.0032	0.0039	0.0002
	CSCTDM	Cure/Survive with TDM	0.9965	0.9969	0.9962	0.0002
	FCCTDM	Failure/Cure with TDM	0.7859	0.7073	0.8645	0.0401
	FMCTDM	Failure/Death with TDM	0.2141	0.2927	0.1355	0.0401
WITHOUT TDM	ICSTDM	Critical infection/Cure without TDM	0.4710	0.4239	0.5181	0.0240
	IMSTDM	Critical infection/Death without TDM	0.2760	0.2484	0.3036	0.0141
	IFSTDM	Critical infection/Failure without TDM	0.2530	0.3277	0.1783	0.0381
	CMSTDM	Cure/Death without TDM	0.0035	0.0032	0.0039	0.0002
	CSSTDM	Cure/Survive without TDM	0.9965	0.9969	0.9962	0.0002
	FCSTDM	Failure/Cure without TDM	0.7859	0.7073	0.8645	0.0401
	FMSTDM	Failure/Death without TDM	0.2141	0.2927	0.1355	0.0401
Analysis 3 Takahashi et al. (2023)						
WITH TDM	ICCTDM	Critical infection/Cure with TDM	0.5854	0.5268	0.6439	0.0299
	IMCTDM	Critical infection/Death with TDM	0.2686	0.2418	0.2955	0.0137
	IFCTDM	Critical infection/Failure with TDM	0.1460	0.2314	0.0606	0.0436
	CMCTDM	Cure/Death with TDM	0.0035	0.0032	0.0039	0.0002
	CSCTDM	Cure/Survive with TDM	0.9965	0.9969	0.9962	0.0002
	FCCTDM	Failure/Cure with TDM	0.7859	0.7073	0.8645	0.0401
	FMCTDM	Failure/Death with TDM	0.2141	0.2927	0.1355	0.0401
WITHOUT TDM	ICSTDM	Critical infection/Cure without TDM	0.4252	0.3827	0.4677	0.0217
	IMSTDM	Critical infection/Death without TDM	0.2834	0.2551	0.3118	0.0145
	IFSTDM	Critical infection/Failure without TDM	0.2530	0.3622	0.2205	0.0362
	CMSTDM	Cure/Death without TDM	0.0035	0.0032	0.0039	0.0002
	CSSTDM	Cure/Survive without TDM	0.9965	0.9969	0.9962	0.0002
	FCSTDM	Failure/Cure without TDM	0.7859	0.7073	0.8645	0.0401
	FMSTDM	Failure/Death without TDM	0.2141	0.2927	0.1355	0.0401

SD, standard deviation; MAX, maximum value; MIN, minimum value; TDM, therapeutic drug monitoring.

### 2.3 Model costs

The study was conducted from the perspective of the National Health System (NHS), thus including only direct healthcare costs. The costs considered in the Markov model were as follows: (i) cost of antibiotic treatment (in 1L and 2L); (ii) cost of hospitalization (ICU stay); (iii) cost of monitoring plasma antimicrobial levels (TDM).

The use of the antibiotics meropenem plus linezolid for 1L treatment and ceftazidime-avibactam plus linezolid or ceftolozane-avibactam plus linezolid for 2L treatment of critical patient infection was considered in the base case analysis, according to an expert panel (Table 2). An average duration of 9–12 days was estimated for 1L treatment and 9.5–12 days for 2L, as recommended in the antibiotic data sheets. The mean values of the stay were calculated from the medians using the formula proposed by Hozo et al. (2005). Antibiotic prices (Ex-factory prices) and dosing regimens were obtained from BotPlus web (BotPlus web) and their summaries of product characteristics, respectively. The total cost of antibiotic treatment in 1L and 2L is shown in Table 2.

**TABLE 2:** Cost and duration of antibiotic treatment and unit cost of TDM.

1. Base case: antibiotic treatment cost (BotPlus web)					
Item	Antibiotics	Dose and regimen*	Duration*	EFP 1 vial**	Treatment cost
Treatment 1L	Meropenem	1,000 mg/8 h	9 days	€ 117.86	€ 3,182.22
	Linezolid	600 mg/12 h	12 days	€ 35.77	€ 858.48
				Total	€ 4,040.70
Treatment 2L	Ceftazidime-avibactam	2,000 mg/8 h	9.5 days	€ 115.00	€ 3,277.50
	Linezolid	600 mg/12 h	12 days	€ 35.77	€ 858.48
				Total	€ 4,135.98
	Ceftolozane-avibactam	1,000 mg/8 h	9 days	€ 91.67	€ 2,475.09
	Linezolid	600 mg/12 h	12 days	€ 35.77	€ 858.48
				Total	€ 3,333.57

2. Sensitivity analysis: antibiotic treatment cost (BotPlus web)					
Treatment 1L	Meropenem	1,000 mg/8 h	9 days	€ 117.86	€ 3,182.22
	Vancomycin	1,000 mg/8 h	9 days	€ 6.90	€ 186.30
				Total	€ 3,368.52
Treatment 2L	Ceftazidime-avibactam	2,000 mg/8 h	9.5 days	€ 115.00	€ 3,277.50
	Vancomycin	1,000 mg/8 h	9 days	€ 6.90	€ 186.30
				Total	€ 3,463.80
	Ceftolozane-avibactam	1,000 mg/8 h	9 days	€ 91.67	€ 2,475.09
	Vancomycin	1,000 mg/8 h	9 days	€ 6.90	€ 186.30
				Total	€ 2,661.39
Treatment 2L	Cefiderocol	2,000 mg/8 h	10 days	€ 138.75	€ 8,325.00
				Total	€ 8,325.00

3. TDM cost (Novy et al., 2023; Universidad de Navarra, 2019)					
		Average value	MIN	MAX	SD
		€ 170.66	€ 87.84	€ 204.79	€ 114.62

*In accordance with what is recommended in the medication technical sheets (Hozo et al., 2005; BotPlus web, 2024).

**BotPlus web, 2024.

1L: first-line antibiotic treatment; 2L: second-line antibiotic treatment; EFP: ex-factory price; MAX: maximum value; MIN: minimum value; SD: standard deviation; TDM: therapeutic drug monitoring.

The unit cost per day of stay in the ICU (€1,404.76 ± €47.22 ([€925.49-€1,110.58]) was obtained as an average of the public health prices of the Spanish regions (Table 3). The length of hospital stay in the ICU was obtained from the meta-analysis of Pai Mangalore et al. (2022) (Table 3).

**TABLE 3:** Costs of the ICU stay.

1. Base case							
Markov states	ICU days*			Total cost**			
	Average	MIN	MAX	Average	MIN	MAX	SD
Treatment in 1L	9	7	14	€ 12,642.80	€ 9,833.29	€ 19,666.57	€ 2,508.49
Cure (with TDM)	11.3	9	13.5	€ 15,803.49	€ 12,642.80	€ 18,964.19	€ 1,612.60
Cure (without TDM)	9.5	7.6	11.4	€ 13,345.17	€ 10,676.14	€ 16,014.21	€ 1,361.75
Treatment in 2L (failure, with TDM)	22.5	18	27	€ 31,606.99	€ 25,285.59	€ 37,928.39	€ 3,225.20
Treatment in 2L (failure, without TDM)	22.6	18.1	27.1	€ 31,694.78	€ 25,355.83	€ 38,033.74	€ 3,234.16

2. Sensitivity analysis: considering only beta-lactam antibiotic treatment							
Markov state	ICU days*			Total cost**			
	Average	MIN	MAX	Average	MIN	MAX	SD
Treatment in 1L	9	7	14	€ 12,642.80	€ 9,833.29	€ 19,666.57	€ 2,508.49
Cure (with TDM)	11.5	9.2	13.8	€ 16,154.68	€ 12,923.75	€ 19,385.62	€ 1,648.44
Cure (without TDM)	9.3	7.4	11.2	€ 13,064.22	€ 10,451.38	€ 15,677.07	€ 1,333.08
Treatment in 2L (failure, with TDM)	25.3	20.2	30.4	€ 35,540.30	€ 28,432.24	€ 42,648.36	€ 3,626.56
Treatment in 2L (failure, without TDM)	23.8	19.0	28.6	€ 33,444.41	€ 26,755.53	€ 40,133.29	€ 3,412.69

*Pai Mangalore et al. (2022).

**Calculated from the average cost of the day of ICU, stay, obtained from the public health prices of the Spanish regions: € 1,404.76 ± € 47.22 (€ 925.49-1,110.58). Euros of 2024.

1L: first-line antibiotic treatment; 2L: second-line antibiotic treatment; ICU: intensive care unit; MAX: maximum value; MIN: minimum value; SD: standard deviation; TDM: therapeutic drug monitoring.

The average cost of TDM (€170.66) was obtained from the work of Novy et al. (2023), considering that 61% of patients would have a second TDM sample. The minimum cost of TDM (€87.84) was obtained from a thesis from the University of Navarra (Universidad de Navarra, 2019). An increase of 20% was considered for the maximum value of the TDM (€204.79) (Table 2). All costs were updated with the CPI for 2024.

### 2.4 Sensitivity analysis

A univariate sensitivity analysis was performed for all variables in the model. Three additional sensitivity analyses were also carried out: (i) treatment with vancomycin instead of linezolid in 1L and 2L; (ii) cefiderocol in 2L (Table 2); and (iii) considering the length of stay observed with beta-lactam antibiotics, according to the meta-analysis by Pai Mangalore et al. (2022) (Table 3).

## 3 Results

### 3.1 Analysis 1 (based on Pai Mangalore)

For each patient undergoing TDM, an additional expenditure of €224 in the deterministic analysis and €195 ± €20 in the probabilistic analysis would be obtained compared to the no-TDM option, with a probability of being the optimal option (probability of generating savings) of 39.4% (Table 4) (Pai Mangalore et al., 2022).

**TABLE 4:** Cost analysis results.

	Deterministic	Probabilistic	
Option	Average cost per patient	Mean cost per patient ± SD (95%CI)	Optimal option (generates savings)
Analysis 1 Pai Mangalore et al. (2022)			
With TDM	€ 16,224	€ 16,120 ± € 1,405 (€ 16,033; 16,207)	39.4%
Without TDM	€ 16,000	€ 15,925 ± € 1,385 (€ 15,839; 16,010)	60.6%
With TDM – Without TDM	€ 224	€ 195 ± € 20	-
Analysis 2 Sanz-Codina et al. (2023)			
With TDM	€ 15,710	€ 15,687 ± € 1,444 (€ 15,597; 15,776)	63.5%
Without TDM	€ 15,966	€ 15,988 ± € 1,411 (€ 15,901; 16,076)	36.5%
With TDM – Without TDM	€ −256	€ −301 ± € 33	-
Analysis 3 Takahashi et al. (2023)			
With TDM	€ 15,639	€ 15,623 ± € 1,407 (€ 15,536; 15,710)	79.7%
Without TDM	€ 16,341	€ 16,308 ± € 1,393 (€ 16,221; 16,394)	20.3%
With TDM – Without TDM	€ −702	€ −685 ± € 14	-

SD: standard deviation; 95%CI: 95% confidence interval; TDM: therapeutic drug monitoring.

### 3.2 Analysis 2 (based on Sanz-Codina)

For each patient undergoing TDM, a saving of €256 in the deterministic analysis and €301 ± €33 in the probabilistic analysis would be obtained compared to the no-TDM option, with a probability of being the optimal option (probability of generating savings) of 63.5% (Table 4) (Sanz-Codina et al., 2023). According to the meta-analysis by Sanz-Codina et al. (2023), TDM of vancomycin did not improve mortality (RR = 0.30; 95% CI 0.06-1.37). Vancomycin TDM was also not associated with greater clinical cure (RR = 1.13; 95% CI 0.77-1.64) (Sanz-Codina et al., 2023).

### 3.3 Analysis 3 (based on Takahashi)

A saving of €702 would be obtained, compared to the no-TDM option, in the deterministic analysis, and €685 ± 14 in the probabilistic analysis, with a probability of being the optimal option (probability of generating savings) of 79.7% (Table 4) (Takahashi et al., 2023). According to the meta-analysis by Takahashi et al. (2023), TDM of aminoglycoside did not improve mortality (RR = 1.33; 95% CI 0.61-2.89). Aminoglycoside TDM was also not associated with greater clinical cure (RR = 1.09; 95% CI 0.82-1.44) (Takahashi et al., 2023).

### 3.4 Univariate deterministic sensitivity analysis

The variables that determined the greatest variability of outcome (cost or savings of TDM) in analyses 1 and 2 were the cost of stay in case of cure with or without TDM, the probability of cure with or without TDM, and the probability of death with or without TDM. In analysis 3, savings were found in all analyses performed (Table 5).

**TABLE 5:** Univariate deterministic sensitivity analysis.

Variable Name	Variable Description	Variable Low	Variable Base	Variable High	Low	High
Analysis 1 Pai Mangalore et al. (2022)						
CECT	Stay cost if cured with TDM	€ 12,643	€ 15,803	€ 18,964	€ −865	€ 1,315
CECST	Stay cost if cured without TDM	€ 10,676	€ 13,345	€ 16,014	€ −533	€ 982
ICCTDM	Critical infection/Cure with TDM	0.6207	0.6897	0.7587	€ −453	€ 902
ICSTDM	Critical infection/Cure without TDM	0.5106	0.5673	0.624	€ −402	€ 851
IMSTDM	Critical infection/Death without TDM	0.1927	0.2141	0.2355	€ −155	€ 604
IMCTDM	Critical infection/Death with TDM	0.16	0.1778	0.1956	€ −91	€ 540
CEFST	Stay cost if failure without TDM	€ 25,356	€ 31,695	€ 38,034	€ −48	€ 497
CTDM	Plasma Levels Cost (TDM)	€ 88	€ 171	€ 205	€ 183	€ 242
CE1L	Stay cost 1L	€ 9,833	€ 12,643	€ 19,667	€ 225	€ 225
CMCTDM	Cure/Death with TDM	0.0032	0.0035	0.0039	€ 225	€ 225
CSCTDM	Cure/Survive with TDM	0.9958	0.9965	0.9972	€ 225	€ 225
FCCTDM	Failure/Cure with TDM	0.7073	0.7859	0.8645	€ 225	€ 225
FMCTDM	Failure/Death with TDM	0.0569	0.2141	0.3713	€ 225	€ 225
CMSTDM	Cure/Death without TDM	0.0032	0.0035	0.0039	€ 225	€ 225
CSSTDM	Cure/Survive without TDM	0.9958	0.9965	0.9972	€ 225	€ 225
FCSTDM	Failure/Cure without TDM	0.7073	0.7859	0.8645	€ 225	€ 225
FMSTDM	Failure/Death without TDM	0.0569	0.2141	0.3713	€ 225	€ 225
CDHOSP	Average daily cost of hospitalization	€ 471	€ 589	€ 565	€ 225	€ 225
CDUCI	Average daily cost in ICU	€ 925	€ 1,405	€ 1,111	€ 225	€ 225
CEFT	Stay cost if failure with TDM	€ 25,286	€ 31,607	€ 37,928	€ 225	€ 225
Analysis 2 Sanz-Codina et al. (2023)						
CECT	Stay cost if cured with TDM	€ 12,643	€ 15,803	€ 18,964	€ −1,263	€ 751
CECST	Stay cost if cured without TDM	€ 10,676	€ 13,345	€ 16,014	€ −885	€ 372
ICCTDM	Critical infection/Cure with TDM	0.5733	0.637	0.7007	€ −881	€ 369
ICSTDM	Critical infection/Cure without TDM	0.4239	0.471	0.5181	€ −776	€ 264
IMSTDM	Critical infection/Death without TDM	0.2484	0.276	0.3036	€ −745	€ 233
IMCTDM	Critical infection/Death with TDM	0.2124	0.236	0.2596	€ −674	€ 162
CEFST	Stay cost if failure without TDM	25.356 €	31.695 €	38.034 €	€ −655	€ 143
CTDM	Plasma Levels Cost (TDM)	€ 88	€ 171	€ 205	€ −298	€ −239
CE1L	Stay cost 1L	€ 9,833	€ 12,643	€ 19,667	€ −256	€ −256
CMCTDM	Cure/Death with TDM	0.0032	0.0035	0.0039	€ −256	€ −256
CSCTDM	Cure/Survive with TDM	0.9958	0.9965	0.9972	€ −256	€ −256
FCCTDM	Failure/Cure with TDM	0.7073	0.7859	0.8645	€ −256	€ −256
FMCTDM	Failure/Death with TDM	0.0569	0.2141	0.3713	€ −256	€ −256
CMSTDM	Cure/Death without TDM	0.0032	0.0035	0.0039	€ −256	€ −256
CSSTDM	Cure/Survive without TDM	0.9958	0.9965	0.9972	€ −256	€ −256
FCSTDM	Failure/Cure without TDM	0.7073	0.7859	0.8645	€ −256	€ −256
FMSTDM	Failure/Death without TDM	0.0569	0.2141	0.3713	€ −256	€ −256
CDHOSP	Average daily cost of hospitalization	€ 471	€ 589	€ 565	€ −256	€ −256
CDUCI	Average daily cost in ICU	€ 925	€ 1,405	€ 1,111	€ −256	€ −256
CEFT	Stay cost if failure with TDM	€ 25,286	€ 31,607	€ 37,928	€ −256	€ −256
Analysis 3 Takahashi et al. (2023)						
CECT	Stay cost if cured with TDM	€ 12,643	€ 15,803	€ 18,964	€ −1,627	€ 223
CECST	Stay cost if cured without TDM	€ 10,676	€ 13,345	€ 16,014	€ −1,269	€ −134
ICCTDM	Critical infection/Cure with TDM	0.5268	0.5854	0.6439	€ −1,276	€ −127
ICSTDM	Critical infection/Cure without TDM	0.2551	0.2834	0.3118	€ −1,203	€ −199
IMSTDM	Critical infection/Death without TDM	0.2418	0.2686	0.2955	€ −1,178	€ −227
IMCTDM	Critical infection/Death with TDM	0.3827	0.4252	0.4677	€ −1,171	€ −233
CEFST	Stay cost if failure without TDM	25.356 €	31.695 €	38.034 €	€ −1,163	€ −241
CTDM	Plasma Levels Cost (TDM)	€ 88	€ 171	€ 205	€ −743	€ −685
CE1L	Stay cost 1L	€ 9,833	€ 12,643	€ 19,667	€ −702	€ −702
CMCTDM	Cure/Death with TDM	0.0032	0.0035	0.0039	€ −702	€ −702
CSCTDM	Cure/Survive with TDM	0.9958	0.9965	0.9972	€ −702	€ −702
FCCTDM	Failure/Cure with TDM	0.7073	0.7859	0.8645	€ −702	€ −702
FMCTDM	Failure/Death with TDM	0.0569	0.2141	0.3713	€ −702	€ −702
CMSTDM	Cure/Death without TDM	0.0032	0.0035	0.0039	€ −702	€ −702
CSSTDM	Cure/Survive without TDM	0.9958	0.9965	0.9972	€ −702	€ −702
FCSTDM	Failure/Cure without TDM	0.7073	0.7859	0.8645	€ −702	€ −702
FMSTDM	Failure/Death without TDM	0.0569	0.2141	0,3713	€ −702	€ −702
CDHOSP	Average daily cost of hospitalization	€ 471	€ 589	€ 565	€ −702	€ −702
CDUCI	Average daily cost in ICU	€ 925	€ 1,405	€ 1,111	€ −702	€ −702
CEFT	Stay cost if failure with TDM	€ 25,286	€ 31,607	€ 37,928	€ −702	€ −702

ICU: intensive care unit; TDM: therapeutic drug monitoring.

### 3.5 Additional deterministic sensitivity analyses

Additional analyses (vancomycin instead of linezolid in 1L and 2L, cefiderocol in 2L, and length of hospital stay with beta-lactams) did not change the direction of the results: additional expenditure in analysis 1, savings in analysis 2 and 3 (Table 6).

**TABLE 6:** Additional univariate deterministic sensitivity analysis.

Sensitivity analysis	Strategies	Cost	Incremental cost (With TDM – Without TDM)
Analysis 1 Pai Mangalore et al. (2022)			
Vancomycin instead of linezolid in 1L	With TDM	€ 15,843	€ 253
	Without TDM	€ 15,590	
Cefiderocol in 2L	With TDM	€ 18,670	€ 27
	Without TDM	€ 18,643	
Length of stay with beta-lactams	With TDM	€ 16,461	€ 350
	Without TDM	€ 16,111	
Analysis 2 Sanz-Codina et al. (2023)			
Vancomycin instead of linezolid in 1L	With TDM	€ 15,331	€ −214
	Without TDM	€ 15,545	
Cefiderocol in 2L	With TDM	€ 18,144	€ −545
	Without TDM	€ 18,689	
Length of stay with beta-lactams	With TDM	€ 15,933	€ −189
	Without TDM	€ 16,122	
Analysis 3 Takahashi et al. (2023)			
Vancomycin instead of linezolid in 1L	With TDM	€ 15,303	€ −702
	Without TDM	€ 16,005	
Cefiderocol in 2L	With TDM	€ 15,974	€ −1,036
	Without TDM	€ 17,010	
Length of stay with beta-lactams	With TDM	€ 15,870	€ −666
	Without TDM	€ 16,536	

1L: first-line antibiotic treatment; 2L: second-line antibiotic treatment; TDM: therapeutic drug monitoring.

## 4 Discussion

An analysis of the economic impact of antimicrobial TDM in Spain, compared to no monitoring, was performed considering three scenarios based on three published meta-analyses. According to the meta-analysis considered, TDM would result in an additional expenditure of €195 or a saving of €685 (low costs, below the daily cost of ICU admission), (Dooley et al., 2021), with a saving probability of only 39.4% or 79.7%, respectively. This uncertainty is due to the fact that the three available meta-analyses obtained highly variable results regarding cure rates or ICU stay length. Therefore, it is necessary to analyze the reasons for these discrepancies by comparing the methods and results of these meta-analyses. The cure rate with or without TDM (Table 1) is the variable with the greatest impact on outcome (Table 5). In this respect, the difference between TDM and non-TDM was 12.2% in the meta-analysis by Pai Mangalore et al. (2022), 16.6% in Sanz-Codina et al. (2023), and 16.0% in Takahashi et al. (2023) On the other hand, with TDM, there was a reduction in treatment failure of 8.6%, 12.6% and 14.5%, respectively. The average cost of 1L treatment failure is estimated to be more than €30,000 per patient (Table 3), which would explain the additional expenditure in test 1 and the savings in tests 2 and 3 (Pai Mangalore et al., 2022; Sanz-Codina et al., 2023; Takahashi et al., 2023).

To understand the different results of the meta-analyses, it is necessary to consider the methodological differences between them. There were differences in the type of studies included (RCTs and observational studies in Pai Mangalore, only RCTs in Sanz-Codina and Takahashi), in their number (11, 10 and 5 studies, respectively), in the number of patients with TDM (765, 624 and 510, respectively) (Pai Mangalore et al., 2022; Sanz-Codina et al., 2023; Takahashi et al., 2023). There were also differences in the typology of patients in eligible studies (all adults): critically ill patients with confirmed or suspected sepsis (Pai Mangalore), with infection treated with antibiotics or antifungals in controlled studies of TDM (Sanz-Codina) and critically ill patients with sepsis, admitted to ICU with mechanical ventilation (Takahashi). Meta-analysis heterogeneity (I^2^) was low for the cure rate in Pai Mangalore and high in Sanz-Codina and Takahashi. The risk of bias was high in 3 of the 4 RCTs included in the Pai Mangalore meta-analysis, 6 of 10 RCTs included in Sanz-Codina and 1 of 5 RCTs in Takahashi (Pai Mangalore et al., 2022; Sanz-Codina et al., 2023; Takahashi et al., 2023). Another important difference to highlight is that the meta-analysis by Pai Mangalore included only beta-lactam antibiotics, while the one by Sanz-Codina included vancomycin as well, and the one by Takahashi included vancomycin and aminoglycosides as well. In this regard, it is interesting to note that the meta-analyses that included vancomycin and aminoglycosides obtained better costs results than the meta-analysis that only included beta-lactams.

In conclusion, the differences in the results of the meta-analyses could be explained by their considerable methodological differences and the different studies and antimicrobials included in the meta-analyses. To resolve this uncertainty about the effectiveness of antimicrobial TDM, it would be desirable if a future multicenter, randomized, controlled clinical mega-trial could be conducted in a well-defined patient population. In addition to the uncertainty derived from meta-analyses, this model has the inherent limitations of a theoretical model, which is nevertheless a useful simulation of clinical reality (Rubio-Terrés et al., 2004). Finally, it may also be considered a limitation that part of the length of ICU stays considered in the model may not be attributable to infection but to other concomitant diseases.

A few studies have been published exploring the economic impact of antimicrobial TDM, but not in critical patients. According to a retrospective study published by Pea et al. (2016), clinical pharmacological advice based on TDM results could lead to reductions in the dose of linezolid, achieving considerable savings. Regarding the TDM of aminoglycosides, as established in the study by Slaughter and Cappelletty (1998), monitoring of aminoglycosides would be economically justified in patients with high rates of nephrotoxicity.

Data from a study of critically ill patients showed an increase in costs related to the management of critically ill patients who underwent therapeutic drug monitoring of beta-lactams (Edwolt et al., 2021). Based on the available evidence, it appears that TDM of beta-lactams is associated with higher costs without these costs being translated into an impact on the mortality of critically ill patients.

## 5 Conclusions

In critically ill patients undergoing TDM of antimicrobial therapy, there is an increased likelihood of cure. However, currently available data are not conclusive on the economic impact of such a therapeutic effect. More reliable clinical data on the effectiveness of TDM are needed, along with economic studies to help determine its efficiency in clinical practice (Pai Mangalore et al., 2023). In the current health situation, with the aim of defining the type of antibiotic and critical patient that could benefit from this strategy, it seems that TDM should be conceptualized in the research area rather than being introduced into routine clinical practice, with the exception of aminoglycoside antibiotics and vancomycin, in which case TDM is fully justified by their narrow therapeutic index, and nephrotoxicity (Rybak et al., 2020; Roberts et al., 2012).

## Data Availability

The raw data supporting the conclusions of this article will be made available by the authors, without undue reservation.
