# Supplementary material for: Economic impact of individualized antimicrobial dose optimization in the critically ill patient in Spain
Source: Front Pharmacol. 2025 Feb 26;16:1506109. doi: 10.3389/fphar.2025.1506109 (PMC11897268; doi:10.3389/fphar.2025.1506109)
Supplement: Supplementary file 1 [file DataSheet1.pdf]

FRONTIERS IN PHARMACOLOGY

Economic impact of individualized antimicrobial dose optimization in the critically ill patient in Spain

SUPPLEMENT 1

What is a Markov model?

A Markov model applied to the healthcare field simulates the evolution of a disease assuming that the patient is always in one of a finite number of health states (for example, health, illness or death). These health states must be exhaustive and mutually exclusive. Health states can be of two types: absorbing (those that cannot be abandoned, the most common and obvious being the state of death) and non-absorbing (any state from which one can pass to another) [1].

In the present study, five health states were considered: first-line treatment (1L) (the state in which the entire patient cohort starts the simulation), cure, second-line treatment-failure (2L), hospital discharge and death (Figure 1).

How does a Markov model work? An example step-by-step

Markov cost model calculations are based on two main parameters: the transition probabilities between states and the costs of each state.

1. *Transition of patients between the states of the model:*

We see the example of the patient with TDM/MIDP and in the 1L treatment state. The evolution of patients with and without TDM to the progression states of cure, failure, survival and death was modeled. For example, a patient in the TDM group who is treated in 1L can evolve in three different ways: cure, failure or death. The probability of cure (ICCTDM with TDM in Figure 1) is indicated in Table 1 (Transition probabilities of the economic model) and is 0.6897 +/- 0.0352 (this probability was obtained from the meta-analysis of Pai Mangalore, 2022). The probability of death (IMCTDM in Table 1) was obtained from the same source. The probability of failure is the complementary (1- (probability of cure + probability of death)).

2. *Cost per patient for each Markov state:*

We follow the same example, looking at the patient with TDM/MIDP and in the 1L treatment state. The cure in 1L has a cost for the patient's stay in the ICU, derived from the length of hospital stay obtained in the corresponding meta-analyses. In this state of health, two other costs occur: the cost of antibiotic treatment (Table 2) and the cost of determining plasma levels (in the case of the TDM group). For example, the average cost of the stay in the ICU is € 12,642.80 (see Table 3).

3. *Deterministic Analysis Calculations*

The cost of each state is obtained by multiplying the cost of each outcome by its corresponding probability. For example, in the deterministic analysis the average cost per patient of the option with TDM is € 16,224 (Table 4).

4. *Probabilistic Analysis Calculations*

Costs and probabilities have a variability given by their standard deviations. This uncertainty is analyzed using probabilistic analysis. Probabilistic analyses were performed using second-order Monte Carlo simulations with 1,000 iterations. The costs were adjusted to gamma distributions and probabilities to beta distributions. Parameters α and λ of the gamma distributions and α and β of the beta distributions were obtained from the means and standard deviations of each variable using the method of moments (see references in the text of the article). In this way, mean values, their 95% CIs and the probability of savings with one or the other option were obtained.

5. Univariate deterministic sensitivity analysis

Univariate sensitivity analyses were also performed, in which only one variable was modified in each analysis (Table 5).

References

1. Rubio Terrés C, Rubio Rodríguez D. Glosario de Farmacoeconomía. Available at URL: https://www.healthvalue.org/pdfs/GLOSARIO%20DE%20FARMACOECONOM%C3%8DA.pdf (access: 15/01/2025).
